# Spot on for liars! How public scrutiny influences ethical behavior

**DOI:** 10.1371/journal.pone.0181682

**Published:** 2017-07-17

**Authors:** Andreas Ostermaier, Matthias Uhl

**Affiliations:** 1 TUM School of Management, Technische Universität München, Munich, Germany; 2 TUM School of Governance, Technische Universität München, Munich, Germany; Middlesex University, UNITED KINGDOM

## Abstract

We examine whether people are more honest in public than in private. In a laboratory experiment, we have subjects roll dice and report outcomes either in public or in private. Higher reports yield more money and lies cannot be detected. We also elicit subjects’ ethical mindsets and their expectations about others’ reports. We find that outcome-minded subjects lie less in public to conform with their expectations about others’ reports. Ironically, these expectations are false. Rule-minded subjects, in turn, do not respond to public scrutiny. These findings challenge the common faith in public scrutiny to promote ethical behavior. While public scrutiny eventually increases honesty, this effect is contingent on people’s mindsets and expectations.

## 1 Introduction

Some say that ethics starts with asking yourself whether you would want to see what you do reported in the newspaper. The intuition for this ethical rule of thumb is straightforward: what you do in private cannot be ethical unless it stands up to public scrutiny. It is not obvious, though, whether people really act more ethically in public than in private. Empirical evidence on the effect of public scrutiny on ethical behavior is surprisingly scant. As a step toward closing this gap, we examine the impact of public scrutiny on lying, which is considered unethical in most cultures [1].

Ethical behavior is contingent on empirical expectations about others’ behavior [2]. People first form expectations and then potentially conform to these expectations. We conduct a laboratory experiment to examine both steps. Specifically, we have subjects roll dice and report their outcomes to earn money. The experiment invites subjects to lie because higher reports yield more money and reports cannot be verified [3]. To study the effect of public scrutiny, we manipulate whether these unverifiable reports are made in public or in private. Before rolling dice, subjects are required to state their expectations about others’ reports.

After reporting, subjects take a test that elicits their ethical mindset and allows us to categorize them as either outcome-minded or rule-minded. The mindset is an important predictor of ethical behavior [4]. Rule-minded individuals feel committed to follow rules per se. Outcome-minded individuals, in turn, condition their behavior, including whether they follow some rule, on its consequences [5]. For example, they consider that lying in public, as opposed to lying in private, may attract negative attention or induce others to lie. Outcome-minded subjects are therefore arguably more susceptible to public scrutiny than rule-minded subjects.

The experimental setup protects liars from being caught individually, whether reports are made in public or in private. However, we can infer lies on the group level. We can thus observe, on the group level, whether subjects lie, whether they expect that others lie, and whether they conform to their expectations. In particular, we can investigate the impact of public scrutiny on honesty, expectations about others’ honesty, and conformity with these expectations. While the emergence and robustness of these expectations is an intriguing issue in itself, we focus on the effect of expectations on behavior rather than of behavior on expectations.

## 2 Theory

Expectations about others are crucial in shaping ethical behavior. People will not conform to some norm unless they expect others to conform to it as well [2]. In particular, people have been found to condition their level of lying on how much they believe others lie, and they adjust their level of lying when their beliefs turn out wrong [6, 7]. While there are prescriptive norms saying that one ought to tell the truth, it is common knowledge that lies are frequent [8], and this observation informs expectations about how much others lie in a given situation. People will therefore expect others, on average, neither to tell the truth nor to lie to the maximum extent when these have to choose between being honest or dishonest.

It is less clear, though, whether expectations about others’ honesty differ in public and private. While we are unaware of direct evidence, it is interesting to note that transparency is called for in all realms of life to promote ethical behavior. These calls for transparency apparently reflect the expectation that people act more ethically in public, which includes that they are more honest. Specifically, the prospect of public scrutiny has arguably a similar effect as situational cues that increase the salience of norms [9, 10]. Although public scrutiny does not directly remind people to tell the truth, it reminds them that ethical behavior is in order. Prior research also shows that people are reluctant to lie in fact-to-face communication [11].

Related research on honesty in groups offers mixed evidence. On the one hand, several studies suggest that people lie more in groups [12]. Explanations include lack of accountability, peer influence, and shared interest [12–14]. In particular, communication offers opportunities to exchange arguments that justify dishonesty [12]. However, this evidence is based on anonymous interaction in the laboratory. Experiments with familiar peers, on the other hand, show that people are more honest in the presence of in-group peers than alone [15]. Taken together, this mixed related evidence seems to justify the conservative prediction that expectations about honesty are at least as high in public—i.e., face-to-face with peers—as in private.

Given their expectations about others’ behavior, people may conform to these expectations even when this is not in their immediate self-interest [2, 16]. For example, economic experiments offer evidence that people forgo monetary gains to follow fairness or reciprocity norms, albeit in total anonymity [17]. This said, an important motivation for conformity is to garner social approval or avoid rejection [18, 19]. While non-conformity may be psychologically costly in private, it is certainly more costly in public, where it attracts direct negative attention. In addition to the psychological cost, lying in public is also costly from an ethical viewpoint. Liars give others a bad example, which these may imitate and thus spread unethical behavior.

It is important to note, however, that behavior depends heavily on ethical mindsets. The distinction between outcome-based and rule-based mindsets has proven particularly helpful in predicting ethical behavior [4]. Rule-minded individuals feel obliged to conform to rules per se, regardless of the specific situation [5, 20]. Outcome-minded individuals, by contrast, consider the consequences of what they do, such as attracting negative attention or giving a bad example to others [5, 21]. This makes them responsive to situational factors, including public attention. Hence, the argument that public scrutiny leads to conformity with empirical expectations about others holds for outcome-minded much more than for rule-minded people.

To illustrate this intuition, imagine a pedestrian who is about to run the light [15]. An outcome-minded pedestrian will consider whether other people and especially children are around. He will have no qualms about running the light per se, but he will be ashamed to be seen breaking the law and refrain from offering a bad model to children, who may be crushed by a car when following his example. A rule-minded pedestrian will stop because it is the law, whether or not others see him and possibly follow his example. Rule-minded individuals are consistent in their ethical behavior, while outcome-minded individuals respond differently to different situations, which also explains why they engage in moral balancing [4].

## 3 Experiment

### 3.1 Ethics statement

The experiment was approved by the Institutional Review Board of experimenTUM (social science lab of Technische Universität München). The investigation was conducted according to the principles expressed in the Declaration of Helsinki. Written consent was obtained from the participants.

### 3.2 Overview

To examine the effect of public scrutiny on honesty, we conducted the dice experiment, which allows subjects to lie without any risk of being caught [3, 6]. The subjects were placed in separate booths, where they were isolated from each other and could not be observed. They rolled a six-sided die and reported their outcome. The setup encouraged lying because pay increased with the reported outcome and the reports could not be verified on the individual level. However, we can infer dishonesty on the group level. We manipulated, between subjects, whether outcomes were reported in public or in private. The laboratory offers a highly controlled environment to investigate the impact of public scrutiny on honesty, expectations, and conformity with expectations.

After entering the laboratory, subjects received full written instructions, which they first read on their own and which were then read out to them. They thus knew that everyone else had received the same information. Each session involved two tasks. First, subjects guessed the outcome to be reported on average by the other subjects in their session. Second, they rolled a die and reported their outcome in private. In the public condition, they then also reported their outcome in public. Next, they answered post-treatment questions, starting with a trolley dilemma to distinguish between outcome-minded and rule-minded subjects. A coin was tossed to determine whether they were paid for guessing or reporting. Subjects were finally paid individually and in cash when leaving the laboratory.

We designed the experiment to allow subjects to form expectations about others and potentially conform to these expectations. We therefore had them first state their expectations (i.e., guess the outcome to be reported on average) and then report their outcomes. Reports can be influenced by expectations, but expectations not by reports. The instructions explained both tasks, starting with the report, which subjects needed to understand to make their guess. They were paid either for their report or their guess rather than both to minimize dependencies between both tasks along with potential hedging or wealth effects. A limitation of this design is that it does not allow subjects to update their expectations, which they can in other experiments [6, 7, 12], nor tell us how robust these expectations are.

Subjects were all students from various disciplines, who knew each other enough to form sound expectations about average peer behavior [15]. Student samples are commonly used to gain insights into social and ethical behavior [22]. Subjects were randomly assigned to one of the two conditions. All sessions had thirteen participants. We thus created constant conditions across sessions and facilitated the guessing task, as the number of other subjects is a multiple of six. A tailor-made graphical computer interface was used for communication [23]. (See S1 File for the written instructions.)

### 3.3 Guessing task

The subjects made an incentivized guess of the outcome that all other subjects in their session would report on average. With thirteen subjects in each session, the reports of the other twelve subjects would average 3.5, if these reported their outcomes truthfully. Each subject’s pay for guessing depended on the accuracy of his or her guess. Table 1 lists the pay-off for each level of accuracy.

**Table 1:** Payoff for guess.

Deviation	Payoff
± 0.1	€12
± 0.2	€10
± 0.3	€ 8
± 0.4	€ 6
± 0.5	€ 4
> ± 0.5	€ 2

Before subjects entered their guesses into their computers, the instructions reminded them that the average report would be 3.5 if each outcome were reported equally often, i.e. twice. We thus ensured that participants who were less familiar with statistics had the same information to form expectations for their guess. If recalling the mean created an anchor for subjects, this anchor was the same across conditions.

We had subjects guess the mean rather than the distribution of outcomes reported by the others [3, 12], because we wanted them to think about what might be the empirical norm rather than about what others might do individually. While they arguably thought first about what everyone else would report, they had to aggregate this information on the group level when entering the outcome to be reported on average.

The task was finished when everyone had entered their guess. Dice were distributed to the subjects only then for the second task.

### 3.4 Reporting task

The subjects rolled their dice as often as they wanted to convince themselves that these were not loaded. However, they were asked to retain and report the outcome of their first die roll [3]. Each subject’s pay was his or her reported outcome multiplied by two (i.e. €2, 4,…, 12). The task was finished when everyone had entered their outcome into their computer.

### 3.5 Private and public condition

In the public condition, subjects were asked to stand up and turn face-to-face after entering their outcome. When they stood face-to-face, they were called on in random order to announce their reported outcome loud and clear in this forum. To prevent path-dependency, an experimenter double-checked that everyone announced the same outcome that they had entered before into to their computer. Hence, the subjects could not adapt their report ex post in response to the others’ reports.

Once all subjects had publicly reported one by one, they went on to answer post-treatment questions. In the private condition, they moved to the questions immediately after entering their report into their computers, without any public announcement.

### 3.6 Categorization by mindset

The post-treatment questions started with the standard trolley problem [24]. The trolley problem is a classical ethical dilemma, which is widely used to determine people’s ethical mindset [4, 25, 26]. The dilemma reads as follows: “A trolley is out of control and threatens to run over five people. By hitting a switch, the trolley can be diverted to another track. Unfortunately, there is another person on that track. Is it permissible (by hitting the switch) to take the loss of a person’s life to save the life of five people?”

The subjects had to select one of two answers: “Yes, it is permissible (by hitting the switch) to take the loss of a person’s life to save the life of five people,” or “No, it is not permissible (by hitting the switch) to take the loss of a person’s life to save the life of five people.” The subjects who answered yes were categorized as outcome-minded, while those who answered no were categorized as rule-minded.

## 4 Results

We used ORSEE to recruit 130 subjects for the experiment [27]. The subjects’ age averaged 21.6 years (SD 3.0). 60 percent of the subjects were male. 65 subjects were randomly assigned to the private condition, 65 to the public condition. Drawing on prior research, we expected that about 60 percent of our subjects would turn out to be outcome-minded [4, 26]. In fact, 78 subjects, or 60 percent, identified themselves as outcome-minded. Equal numbers of outcome-minded subjects ended up in the public and private conditions. (See S2 File for the data.)

The experiment created a situation where subjects had to choose whether to be honest or dishonest. We have argued that people will expect others neither to tell the truth nor to lie as much as possible in such a situation. In line with this argument, the average outcomes that the subjects expected others to report—4.18 in the public and 4.27 in the private condition—differ both from the average outcome of 3.5 under truthful reporting and from the maximum report of 6.0. An ANOVA of expectations for the effects of public scrutiny and the mindset reveals that these differences are significant (*F*_1,126_ = 53.25, 266.46, *p* < 0.001 in public; *F*_1,126_ = 41.65, 294.69, *p* < 0.001 in private).

For lack of a compelling theory to derive a prediction of whether expectations differ between the public and private condition, we have conjectured that people expect at least as much honesty in public as in private. This argument is mainly based on the observation that transparency—and thus public scrutiny—is often implied to promote ethical behavior. In fact, subjects expect marginally more dishonesty in private than in public (4.18 < 4.27), but expectations do not differ significantly (*F*_1,126_ = 0.36, *p* = 0.552), which is in line with our conservative conjecture. Hence, we note the following result.

**Result 1**. People expected others to lie both in public and private. They expected the same level of dishonesty in both cases.

**Fig 1:**
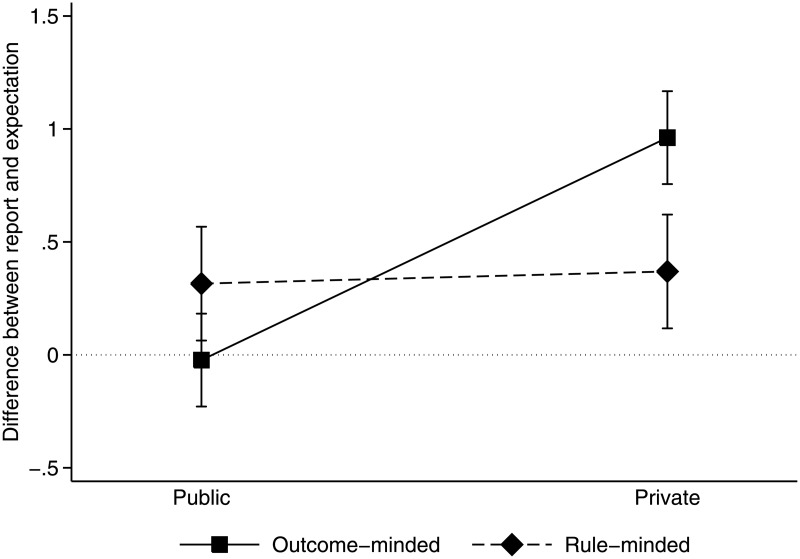
Difference between reports and expectations by condition and mindset.

To test whether subjects conformed to their empirical expectations about others more readily when reports were made in public, we compare the differences between reports and expectations. A small difference indicates that the subjects’ reports matched their expectations about others’ reports. Since we have argued that subjects’ conformity depends on their ethical mindset, we break down these differences by mindsets. Both in the public and private condition, 39 subjects were categorized as outcome-minded and 26 as rule-minded. The differences between reports and expectations are depicted in Fig 1.

From the figure, it is striking that the outcome-minded subjects’ reports perfectly matched their expectations about others in the public condition. The results of an ANOVA and linear post-estimations show that the difference of −0.02 does not differ from zero (*F*_1,126_ = 0.01, *p* = 0.911). In the private condition, by contrast, subjects’ reports exceeded their expectations by 0.96 (*F*_1,126_ = 22.04, *p* < 0.001). Comparing these differences, we find that conformity is significantly higher in public than in private (−0.02 < 0.96, *F*_1,126_ = 11.55, *p* < 0.001).

Contrarily, the rule-minded subjects’ conformity did not differ between the public and private conditions (0.32 < 0.37, *F*_1,126_ = 0.02, *p* = 0.880). While their reports exceeded their expectations, the differences are not significant (*F*_1,126_ = 1.58, *p* = 0.211 in public and *F*_1,126_ = 2.17, *p* = 0.144 in private), which implies conformity in both conditions. Considering the difference in the differences, or the interaction in Fig 1, the conformity of outcome-minded subjects changes significantly more than that of rule-minded subjects, which does not change indeed (*F*_1,126_ = 4.41, *p* = 0.038).

**Result 2**. Only outcome-minded people conformed to their expectations about how much others lie in public more than in private.

**Fig 2:**
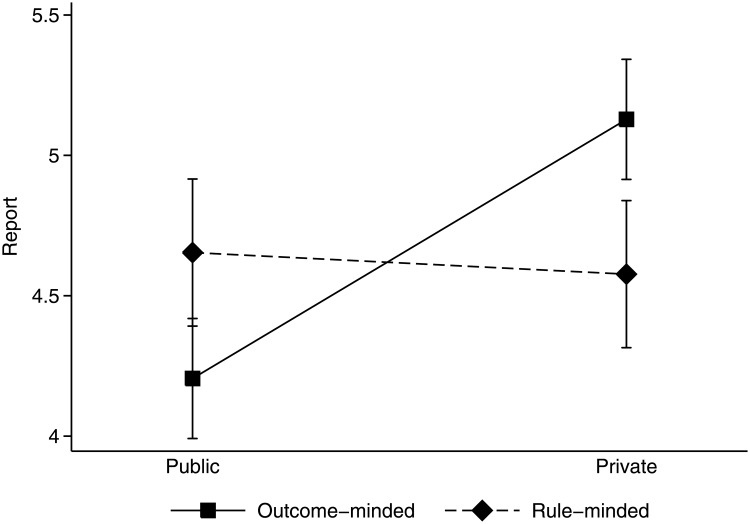
Reports in public and private by mindset.

Recollecting our findings, we note that expectations about others’ honesty were the same across conditions. However, subjects differed in how they conformed to these expectations depending on their mindsets and on whether reports were submitted in public or in private. We have started with the question, though, whether people are more honest in public than in private. To answer this question, we compare subjects’ reports for either mindset, which are depicted in Fig 2, between conditions. The figure shows that outcome-minded subjects’ public reports are much more honest than their private reports, and the results of an ANOVA of the reports show that the difference is significant (4.21 < 5.13, *F*_1,126_ = 9.39, *p* = 0.003). Rule-minded subjects’ reports, in turn, do not differ between conditions (4.65 > 4.58, *F*_1,126_ = 0.04, *p* = 0.835).

All reports were significantly above 3.5, which statistically results in case of truthful reporting. Outcome-minded subjects’ low public reports of 4.21 came closest to this threshold (*F*_1,126_ = 10.96, *p* = 0.001), their high private reports exceeded it most (*F*_1,126_ = 58.43, *p* < 0.001). The rule-minded subjects’ reports also exceeded the threshold in both conditions (*F*_1,126_ = 19.56, *p* < 0.001 in public and *F*_1,126_ = 17.04, *p* = 0.006 in private). Although we think of rule-minded subjects as observing prescriptive rules (such as not to lie or not to run the light in our example), compliance with rules is not a privilege of either one mindset. Across conditions, the outcome-minded and rule-minded subjects’ reports were equal (4.67 > 4.62, *F*_1,126_ = 0.05, *p* = 0.830). Hence, outcome-minded and rule-minded subjects were, on average, equally (dis)honest.

To complement these results, we consider honesty and conformity across mindsets. The overall difference between reports and expectations was 0.11 (SE 0.16) in the public condition and 0.72 (SE 0.16) in the private condition. The former difference does not significantly differ from zero, indicating conformity between reports and expectations in public (*F*_1,126_ = 0.50, *p* = 0.480). In private, however, the subjects’ reports did not match their expectations (*F*_1,126_ = 4.47, *p* = 0.037). Turning to honesty, reports average 4.38 (SE 0.18) in public and 4.91 (SE 0.15) in private. Reports are therefore lower in public than in private, and significantly so (*F*_1,126_ = 5.03, *p* = 0.027).

Hence, the answer to the question whether people are more honest in public than in private is positive. This result, however, is driven by outcome-minded subjects, who respond to public scrutiny, while rule-minded subjects do not. Nonetheless, if the portion of outcome-minded subjects is as substantial as experiments with the trolley problem suggest, the effect of public scrutiny seems to be large enough to increase overall honesty.

## 5 Conclusion

We set out to investigate the common belief that public scrutiny promotes ethical behavior. This belief is reflected, for example, in the popular newspaper test, as much as in common calls for transparency. Specifically, we conducted a laboratory experiment to examine whether people are more honest in public than in private. We find that some people are more honest in public, in order to conform with their expectations about others’ honesty. Our results therefore show that public scrutiny reinforces the effect of empirical expectations, which are crucial in shaping ethical behavior [2, 16]. While our findings thus offer support for the faith in public scrutiny, they also highlight its contingencies.

Specifically, whether people conform with their expectations about others’ behavior is contingent on their mindset. Public scrutiny had a large effect on the outcome-minded subjects, who matched their expectations in public but lied much more than they expected others to lie in private. The rule-minded subjects, by contrast, were unimpressed by public scrutiny; their reports differed hardly in public and in private, slightly exceeding their expectations about others in both cases. Hence, public scrutiny can enhance ethical behavior provided that a large portion of the population is outcome-minded. Variation in the prevalence of ethical mindsets may also shed new light on evidence from cross-cultural studies on honesty [11].

The private condition revealed the intriguing insight that the very people who were susceptible to public scrutiny systematically overestimated others’ honesty or, put differently, allowed themselves transgressions but believed that others would not. This bias was hidden in public, where subjects conformed to their false expectations. Similar biases have been observed in other contexts, where people, on average, consider themselves better or worse than the average [28]. Nonetheless, it is ironic to note that public scrutiny results in more honesty because it leads people to conform with false expectations about others’ honesty. It thus turns the expectation of honesty into an—ethically desirable—self-fulfilling prophecy.

This finding points out another contingency. As public scrutiny reinforces conformity with empirical expectations, it can promote unethical as much as ethical behavior. Imagine an individual who has a conditional preference to conform with some prescriptive norm, but falsely expects that others do not share this preference. Conformity with this false expectation will lead that individual to act unethically despite her preference. Pessimistic expectations may also be used for motivated reasoning to justify one’s own unethical behavior [29]. Many would rather be the crook than the sucker [2, 30]. Prior evidence also argues for an asymmetry in how people respond to ethical and unethical behavior [6], which may extend to expectations about others’ behavior.

This study focuses on the effect of (potentially false) expectations about others on honesty, and especially the impact of public scrutiny on this effect. Expectations, however, depend on behavior as much as behavior depends on expectations. For example, if we had subjects first report and then state their expectations after experiencing the temptation of dishonesty, these might be more accurate. It is still noteworthy that (outcome-minded) subjects formed false expectations although they had full information from the beginning. This said, it is a limitation of this experiment that it teaches us nothing about the emergence, adjustment, and robustness of expectations, which permits interesting extensions of this research.

In summary, we find that public scrutiny actually promotes ethical behavior, but this effect is not as straightforward as one might assume. We believe that our insights warrant further research on the ethical effects of public scrutiny or transparency. Given the key role of conformity, adverse effects are certainly a promising avenue. Economics students, for example, were found to be more generous in dictator games when they were paid in private than in public, probably because they wanted to conform to peers’ expectations of “rational behavior” [31]. Likewise, it is conceivable that public scrutiny erodes honesty when people, contrary to what we see in our study, overestimate others’ dishonesty conform to these pessimistic expectations.

## Supporting information

S1 File Instructions. Written instructions for the experiment.
(PDF)

S2 File Data. Data of the experiment as comma-separated values.
(CSV)
