# Supplementary material for: Spot on for liars! How public scrutiny influences ethical behavior
Source: PLoS One. 2017 Jul 17;12(7):e0181682. doi: 10.1371/journal.pone.0181682 (PMC5513494; doi:10.1371/journal.pone.0181682)
Supplement: S1 File — Written instructions for the experiment. (PDF) [file pone.0181682.s001.pdf]

Instructions

General Instructions

Please keep calm and follow the experimenter's instructions during the experiment. Note that experimenters will never deceive you in experiments conducted in this laboratory.

Please don't talk unless you are told to. If you have any questions, raise your hand. The experimenter will come to you and answer your questions in private. Switch off your mobile devices and stow them in the pocket next to you.

Participants who don't follow the instructions will be excluded from the experiment and will receive only a fixed compensation of €2.

Tasks

The experiment consists of two tasks: the guessing and the dice task. You will be paid for one of these two tasks. A coin will be tossed to determine the paying task at the end of the experiment.

You will first perform the guessing task and then the dice task. However, the guessing task builds on the die task. The dice task will therefore be explained first.

Dice Task

You will receive a six-sided die soon. You roll the die repeatedly to convince yourself that it isn't loaded.

You retain the outcome of your first die roll. You enter the outcome of your first die roll into your computer.

Your pay is *twice the outcome you enter.* Your pay is therefore as follows:

Outcome	Payoff
1	€ 2
2	€ 4
3	€ 6
4	€ 8
5	€10
6	€12

Guessing Task

You guess the *average of the outcomes reported* by the *other twelve* participants in this room. You enter your guess into your computer.

Please round to one decimal place. For instance, if you expect that each number is entered equally often (i.e., twice), you enter 3.5.

The better your guess of the average of the outcomes entered by the other participants, the more you earn. Your pay is determined as follows:

Deviation	Payoff
$\pm$ 0.1	€12
$\pm$ 0.2	€10
$\pm$ 0.3	€ 8
$\pm$ 0.4	€ 6
$\pm$ 0.5	€ 4
$> \pm$ 0.5	€ 2

Establishing Outcomes[1]

All participants stand up and turn face-to-face. The numbers of your workstations are called in random order.

When your number is called, you announce your outcome loud and clearly. The experimenter double-checks that the outcome that you announce is the same as the outcome you have entered into your computer.

Payment

A coin is tossed to determine whether you are paid for the guessing task or for the dice task.

In addition to your payoff of €2–12 from the experiment, you receive a fixed payoff of €2. Hence, you earn at least €4 overall.

You receive your payoff at the end of the experiment in return for the card with the number of your workstation. You are paid confidentially in cash. You are called one by one to the reception room.

[1] The Section "Establishing Outcomes" appeared only in the instructions for the public condition. The instructions were otherwise identical for the public and private condition.
